# The Role of Axial Length-Corneal Radius of Curvature Ratio in Refractive State Categorization in a Nigerian Population

**DOI:** 10.5402/2011/138941

**Published:** 2011-07-13

**Authors:** Eghosasere Iyamu, Joy Iyamu, Christian Izuchukwu Obiakor

**Affiliations:** ^1^Department of Optometry, Faculty of Life Sciences, University of Benin, Benin City, Nigeria; ^2^Eye Clinic, Faith Medical Complex, Benin City, Nigeria

## Abstract

The aim of this study was to investigate the association of axial length (AL)/corneal radius of curvature (CRC) ratio (AL/CRC) with spherical equivalent refractive state (SER) in young adults. A total of seventy (*n* = 70) subjects consisting of 31 males and 39 females participated in this study. Subjects were categorized into emmetropia, hyperopia and myopia using the spherical equivalent refraction. The axial length was measured with I-2100 A-Scan ultrasonography/Biometer (CIMA Technology, USA), the corneal radius of curvature with Bausch & Lomb H-135A (Bausch & Lomb Corp., USA), and the refractive state by static retinoscopy and subjective refraction. The mean AL, CRC and AL/CRC ratio of all subjects were 23.74 ± 0.70 mm, 7.84 ± 0.19 mm, and 3.03 ± 0.14, respectively. Myopes had significantly longer AL, steeper CRC and higher AL/CRC ratio than the emmetropes and hyperopes. There was statistically significant inverse correlation between AL and CRC (*r* = −0.53, *P* < 0.0001), SER (*r* = −0.64, *P* < 0.0001), and between SER and AL/CRC (*r* = −0.78, *P* < 0.0001). A significant positive correlation was found between CRC and SER (*r* = −0.69, *P* < 0.0001). The categorization of the refractive state of an individual is better done by using the AL/CRC ratio index.

## 1. Introduction

The refractive state of the eye is determined by refractive components (corneal power, lens power, anterior chamber depth, and axial length) which are interdependent rather than independent variables, and that the eye grows during the early years in life in such a manner that the refractive state tends towards emmetropia [1, 2]. The refractive state of the human eye is dependent on the balance of change in overall eye size and refractive components, namely, the cornea and crystalline lens [3]. The axial length (AL) is the distance from the corneal surface to an interference peak corresponding to the retinal pigment epithelium/Bruch's membrane [4, 5], and this is expressed in millimeters. Majority of eye growth takes place in the first 18 months of life after which there is little change [6]. Overall the changes in axial length appear to outweigh the progressive corneal flattening with age in normal eyes; the majority of axial length elongation takes place in the first three to 6 months of life and a gradual reducing rate of growth over the next two years [7], and by three years the adult eye size is attained [8]. The cornea is the most powerful refracting surface of the optical system of the eye, accounting for two-thirds of the eye's focusing power. Production of a sharp image at the retinal receptors requires corneal transparency and appropriate refractive power. The refractive power of the cornea depends on its curvature and the difference in refractive indexes between it and air [9]. The interaction between axial length and corneal radius of curvature (CRC) has played a major role in the compensatory adjustments of the optical components of the eye towards attaining emmetropic state [8]. The axial length-corneal radius (AL/CR) ratio has been shown to give a better correlation with refractive error than is obtained with axial length alone [8]. The process operating to produce greater frequency of emmetropia than would be expected on the basis of chance alone is termed emmetropization. Emmetropization mechanism is disturbed if degraded visual images reach the retina [10]. The aim of this study is to determine the role of axial length-corneal radius of curvature (AL/CRC) ratio in refractive state categorization in Nigerians. 

## 2. Materials and Methods

This observational, prospective, cross-sectional study was conducted in Optometry clinic at the University of Benin, Benin City, Nigeria over a period of six months (August 2009 to January 2010). All subjects fulfilled the inclusion criteria: no history of corneal infection, or abnormalities, contact lens wear, systemic disease (such as diabetes or rheumatoid arthritis), ocular trauma or surgery. The subjects' intraocular pressure intraocular pressure had to be between 10–21 mmHg. All procedures were approved by the Departmental Research and Ethics Committee (DREC) of the University in accordance with the tenets of Helsinki. The Bausch and Lomb keratometer H-135A (Bausch & Lomb Technology, USA) was used to measure the corneal radius of curvature. Average corneal curvature (AVK) was obtained by the average of the horizontal and vertical corneal curvature. The axial length was measured with I-2100 A-Scan biometer (CIMA Technology, USA), and the average of three readings were calculated as the measured axial length. All measurements were taken between 9 am and 12 noon.

### 2.1. Procedure

For axial length measurement, the subject was comfortably seated with the head upright and eyes in the primary position of gaze. The probe was sterilized with 70% alcohol and allowed to air-dry. A drop of topical anaesthetic (Tetracaine Hcl 0.1%) was instilled in subject's eye. The probe was carefully aligned perpendicularly to and highly applanating the cornea. The axial length is displayed on the colour liquid crystal display (LCD) screen. At least three readings were taken and the average calculated as the measured axial length. 

For keratometry, the eyepiece or reticule was adjusted for the examiner's refractive status. The subject was seated comfortably before the instrument with forehead on the head rest and chin fitting snugly into the chin rest. The leveling sight pin was at the same level as the outer canthus of the eye to be assessed. At this point, the instrument was switched on and the examiner viewed the mire through the eyepiece while patient was asked to fixate on the reflection of his/her own eye. The blurred mire was cleared by adjusting the focusing knob. The cross-hair was placed in the center of the focusing circle to ensure that the optical axis of the instrument was coincident with the visual axis of the patient to ensure accuracy of readings by adjusting the elevation knob. Once the exact position was obtained the lock knob was tightened so that the instrument does not rotate out of setting. The minus signs are superimposed by the vertical power drum and the plus signs by the horizontal power drum. Three measurements were taken, and the average values for vertical and horizontal corneal curvature were recorded along the appropriate meridians. The average of both values was recorded as the average corneal curvature (AVK). The AL/CRC ratio for each subject was obtained by dividing the axial length by the corneal radius of curvature. The refractive status was obtained objectively (using Keeler retinoscope-Keeler Instruments Inc., USA) and subjectively (using trial lens set-American Opticals). Spherical equivalent refractive status (SER) values were obtained by adding half the cylindrical component to the spherical component. Categorization was done based on: Emmetropia ≤ ±0.50 DS, Myopia > −0.50 DS and Hyperopia > +0.50 DS.

### 2.2. Data Analyses

The Statgraphics Plus ver., 5.1 (Statpoint Technologies Inc., Warrenton, USA) and SPSS ver., 17 (SPSS Inc., Chicago, ill, USA) for the PC were used for statistical analyses and preparation of figures. Measures of spread including standardized kurtosis and standardized skewness were derived. Normality of distribution of data was determined by the spread and Kolmogorov-Smirnov *Z* test (K-S). The distribution of data was considered normal when the values of the spread lie between −2 and 2, and for K-S, when *P* value is greater than 0.05. One-way analysis of variance (ANOVA) was used to compare the mean axial length-corneal radius of curvature and axial length-corneal radius of curvature ratio across the refractive status groups. The correlation between variables was performed with linear regression analysis. A *P* value of ≤ 0.05 was taken as statistically significant.

## 3. Results

A total of seventy (*n* = 70) subjects with mean age 27.9 ± 5.9 years (range, 20 to 39 years), consisting of 31 males and 39 females.  Table 1 shows the descriptive statistics of the measured variables for all subjects.

### 3.1. Mean AL and the Effect of Age, CRC, and SER on AL

**Figure 1:**
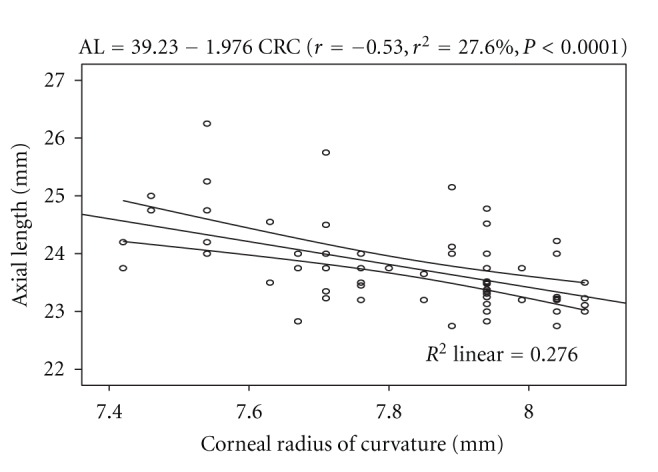
Correlation of axial length and corneal radius of curvature with 95% confidence interval of the regression line.

The Kolmogorov-Smirnov *Z* score for axial length of 1.19 (*P* = 0.12), and the standardized skewness and kurtosis *Z* scores of 1.27 and 1.97 show that axial length was normally distributed. The mean AL of all subjects was 23.74 ± 0.70 mm. The analysis of variance performed on AL shows that the difference in mean AL across the refractive status groups was statistically significant (*F* = 19.6, *df* = 2, 67, *P* < 0.0001). Post-hoc test with Fisher's least significant difference (LSD) revealed that the average AL of myopes was significantly longer than that of emmetropes by 0.80 mm and 0.89 mm longer than that of hyperopes. However, the difference in mean AL between hyperopes and emmetropes was not significant. Table 2 shows the distribution of axial length according to refractive status. Regression analysis performed on axial length and age shows no significant correlation (*r* = −0.056, *r* ^2^ = 0.31%, *P* = 0.65). The linear regression model is represented by AL = 23.98 − 0.081 AGE. However, a statistically significant inverse relationship was found between axial length and corneal radius of curvature (*r* = −0.53, *r* ^2^ = 27.6%, *P* < 0.0001). The linear regression model is represented by: AL = 39.23 − 1.976 CRC. The model as fitted explains 27.6% of the variability in axial length. From the equation, for every decrease of 0.10 mm in corneal radius of curvature (corneal steepening) the axial length is increased by 0.20 mm. This association is represented in Figure 1.

**Figure 2:**
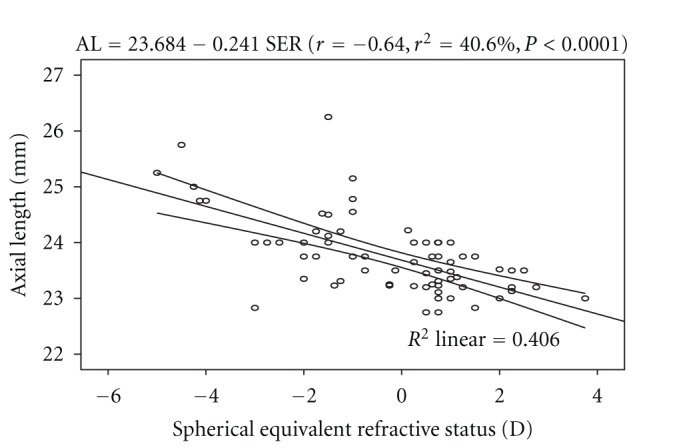
The trend line of the regression of axial length and spherical refractive status with 95% confidence interval of the regression line.

In the same vein, a statistically significant inverse association was found between axial length and spherical refractive status (*r* = −0.64, *r* ^2^ = 40.6%, *P* < 0.0001). The linear regression model is represented by: AL = 23.684 − 0.241 SER. The model as fitted explains 40.6% of the variability in axial length. From the equation representing the model, for every 1.00 D increase in myopia, the axial length is increased by 0.24 mm.  Figure 2 shows the regression model of AL and SER with the 95% confidence interval of the regression line.

### 3.2. Mean CRC: the Effect of Age and SER on CRC

 The Kolmogorov-Smirnov *Z* score of 1.63 and the standardized skewness and standardized kurtosis *Z* score of −0.63 and −0.61 show that corneal radius of curvature values were normally distributed. The mean CRC for all the subjects was 7.84 ± 0.19 mm. ANOVA showed that the difference in mean CRC across the refractive status groups was statistically significant (*F* = 27.9, *df* = 2, 67, *P* < 0.0001). Post-hoc test with Fisher's LSD showed that myopes had steeper corneas than the other two groups (steeper by 0.27 mm than that of hyperopes, and 0.28 mm than that of emmetropes).  Table 3 shows the descriptive statistics of CRC according to refractive status.

**Figure 3:**
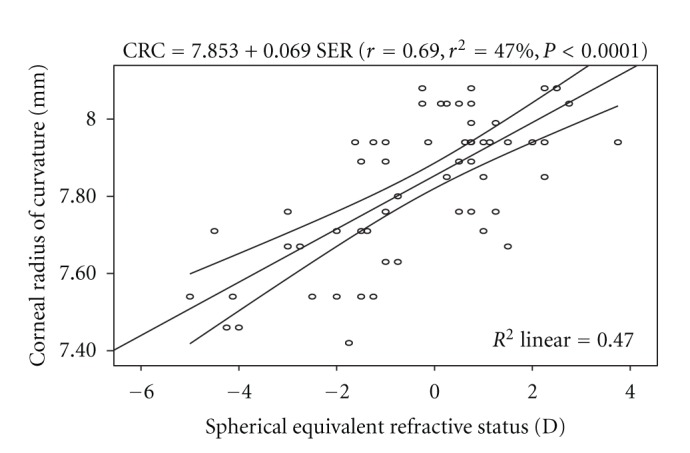
Correlation of corneal radius of curvature and spherical equivalent refraction with 95% confidence interval of the regression line.

Regression analysis performed on axial length and age shows no significant association (*r* = −0.08, *P* = 0.51). The linear regression model is represented by: CRC = 7.948 − 0.004 AGE. However, there was a significant positive correlation between CRC and SER. The linear regression model is represented by: CRC = 7.853 + 0.069 SER. The model as fitted explains 47% of the variability in CRC. From the equation it can be predicted that for every 1.00 D increase in myopia, the cornea is steepened by approximately 0.07 mm.  Figure 3 represent the regression model with the 95% confidence interval of the regression line.

### 3.3. Mean AL/CRC Ratio: Effect of Age and SER on AL/CRC

The kolmogorov-Smirnov *Z* score of 0.93 (*P* = 0.35), and *Z* score of standardized skewness, and standardized kurtosis of 0.81 and −0.25, respectively, show that the values of AL/CRC are normally distributed. The mean AL/CRC ratio was 3.03 ± 0.14. ANOVA showed that the difference in mean AL/CRC ratio between refractive status groups was statistically significant (*F* = 43.12, *df* = 2, 67, *P* < 0.0001). Post-hoc test shows that the mean differences of 0.20 (between myopes and emmetropes) and 0.21 (between hyperopes and myopes) were statistically significant (*P* < 0.05). However, the difference in mean AL/CRC between emmetropes and hyperopes was not significant (*P* > 0.05). The AL/CRC ratio of myopes was much higher than the other two groups. The correlation between AL/CRC ratio and age was not statistically significant (*r* = −0.002, *P* > 0.05). The linear regression model is represented by: AL/CRC = 3.034 − 0.00007 AGE. 

Table 4 shows the descriptive statistics of AL/CRC ratio according to refractive status.

**Figure 4:**
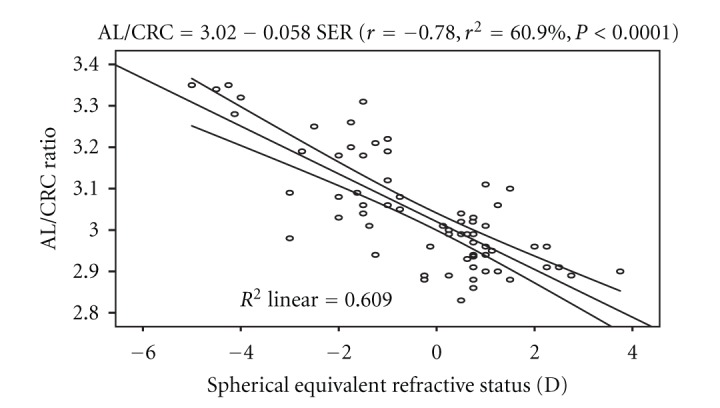
Regression line of the correlation of AL/CRC ratio and SER with the 95% confidence interval of the regression line.

Regression analysis performed on AL/CRC ratio and SER showed a statistically significant inverse correlation (*r* = −0.78, *r* ^2^ = 60.9%, *P* < 0.0001). The linear regression is represented by the equation: AL/CRC = 3.016 − 0.057 SER. The model as fitted explains 60.9% of the variability in AL/CRC ratio.  Figure 4 shows the regression line of the correlation with 95% confidence interval of the regression line.

### 3.4. The Effect of Gender on Measured Variables

The difference in mean AL between males (23.91 ± 0.78 mm) and females (23.60 ± 0.61 mm) was not statistically significant (unpaired *t*-test: *t* = −1.92, *df* = 68, *P* = 0.06). The male showed slightly longer axial length than their female counterparts. This finding was somewhat consistent with the study of Osuobeni [11] who reported that males had significantly longer axial length. Similarly, the difference in mean CRC between males (7.82 ± 0.19 mm) and females (7.85 ± 0.19 mm) was not statistically significant (*t* = 0.68, *df* = 68, *P* = 0.50). Also, the difference in mean AL/CRC ratio between males (3.06 ± 0.14) and females (3.01 ± 0.13) was not significant statistically (*t* = −1.50, *df* = 68, *P* = 0.14).

## 4. Discussion

The cornea has an average radius of curvature of 7.80 mm with an instrument calibrated for index of refraction of 1.3375. The average value of 7.84 ± 0.19 mm obtained from this study can be considered to be same with the average value reported by Waltman and Hart [9]. The axial length has been found to be one of the key variables used in assessing the refractive status of the eye, and the interaction between it and corneal radius of curvature play a major role in the emmetropization process. Numerous studies [3, 8, 9] have shown that axial length and corneal radius of curvature are interdependent variables and that the true refractive state can be assessed based on axial length-corneal radius of curvature index expressed as AL/CRC ratio. In this study, the difference in mean AL across the refractive status groups was statistically significant, with myopes having significantly longer axial length than the other two groups (Table 2). This was consistent with the report of Llorente and colleagues [12] that axial length was significantly higher in myopes than hyperopes. Chen et al. [13] also reported that eyes with more myopic refractive error tended to have greater axial length. Regression analysis performed on AL and age showed no statistically significant association. This was in line with the claim of Tien et al. [14]. Analysis of variance performed on CRC across the refractive status groups showed that the mean difference was statistically significant, with myopes having steeper corneas than the hyperopes and emmetropes (Table 3). The values of AL/CRC ratio obtained from this study are 3.16 (SD 0.12) for myopes, 2.95 (SD 0.07) for hyperopes, and emmetropes had 2.96 (SD 0.07), respectively. These values were comparable with 2.98 (SD 0.69) for emmetropes, hyperopes 2.89 (SD 0.87), low myopes, 3.01 (SD 0.07) and 3.10 (SD 0.11) for moderate myopes reported by Yebra-Pimentel et al. [15]. The difference in mean AL/CRC ratio across the refractive groups was significant, with myopes having higher AL/CRC ratio than emmetropes and hyperopes. This is consistent with the claim of Osuobeni [11] that myopes had significantly higher ratio than nonmyopes. AL/CRC ratio was not affected by age (*r* = −0.002, *P* > 0.05). An inverse relationship was found between axial length and corneal radius of curvature (*r* = − 0.53, *P* < 0.0001). From the linear regression equation (AL = 39.23 − 1.972 CRC) longer axial length is associated with steeper cornea. Chen et al. [13] reported that eyes with axial elongation tended to have flatter cornea (*r* = −0.502, *P* < 0.001). On the other hand, Osuobeni [11] found a positive correlation between axial length and corneal curvature. An inverse correlation was found between AL and SER (*r* = −0.64, *P* < 0.0001) and from the regression model equation (AL = 23.684 − 0.241 SER), a 1.00 D increase in myopia would lead to 0.24 mm increase in axial length. This was consistent with the claim of Chen et al. [13] that eyes with more myopic refractive error tended to have greater axial length (*r* = −.645, *P* < 0.001). The result of this study also shows that there was a statistically significant inverse correlation between AL/CRC ratio and SER (*r* = − 0.77, *P* < 0.0001). The linear regression equation is AL/CRC = 3.016 − 0.0573 SER. A change of 1.00 D in spherical equivalent refractive error will alter the AL/CRC ratio by approximately 0.06. Yebra-Pimentel et al. [15] reported a higher correlation between AL/CR ratio and refractive error. Also, Llorente et al. [12] reported that AL/CR was highly correlated with SER. Ojaimi et al. [16] also reported a high correlation between AL/CR and refractive error. It is important to note that although the subjects in Ojaimi and colleagues' study were children, the result was still comparable to that found in this study with young adults aged between 20 and 39 years suggesting that the statistically significant correlation between AL/CRC and refractive error is true at least among the nonpresbyopes. Intuitively, AL/CRC ratio is a better index of categorizing refractive status even in the black race. Myopes have been shown to have higher AL/CRC ratio than emmetropes and hyperopes. The inverse relationship between axial length and corneal radius of curvature supports the mechanism of emmetropization described by Grosvenor [17]. He asserted that as the axial length increases tending to bring about myopia, the cornea tend to flatten bringing a decrease in myopia. This mechanism brings about a greater frequency of emmetropia than is expected on the basis of chance alone. The inverse correlation between axial length and corneal radius of curvature demonstrates the eye's ability to compensate for normal physiologically driven axial length changes. Although male subjects showed longer axial length than the female counterparts, the difference in mean AL between them was not significant. This was contrary to the claim of Osuobeni [11] that males significantly have longer axial length than females. Gender-related differences in mean CRC and AL/CRC ratio were not statistically significant. 

In conclusion, there was a significant association between axial length-corneal radius of curvature and spherical equivalent refractive state. Also, there was a statistically significant correlation between AL/CRC ratio and SER. AL/CRC ratio is a better index for categorizing the refractive status of an individual than axial length alone even among the black race. 

## Figures and Tables

**Table 1:** Descriptive statistics of measured variables of all subjects.

Statistics	Variables				
	SER (D)	AL (mm)	AGE (years)	AL/CRC	CRC (mm)
Count	70	70	70	70	70
Mean	−0.24	23.74	27.9	3.03	7.84
SD	1.85	0.70	6.00	0.14	0.19
MIN	−5.00	22.75	20.00	2.83	7.42
MAX	+3.75	26.25	39.00	3.35	8.08
Stnd skewness	−0.59	1.27	0.33	0.81	−0.63
Stnd kurtosis	0.05	1.97	−1.23	−0.25	−0.61
K-S (*Z* score)	1.29	1.19	1.20	0.94	1.63
(*P* value)	0.07	0.12	0.11	0.35	0.01
SEM	0.22	0.08	0.71	0.02	0.02
95% CI	−0.02–−0.46	23.66–23.82	27.18–28.60	3.01–3.05	7.82–7.86

SD: standard deviation; Stnd skewness: standardized skewness; Stnd kurtosis: standardized kurtosis; Min: minimum; Max: maximum; K-S: Kolmogorov-Smirnov *Z*; SEM: standard error of mean; 95% CI: 95% confidence interval.

**Table 2:** Descriptive statistics of axial length according to refractive status.

Statistics	Refractive status		
	Emmetropia	Hyperopia	Myopia
Count	11	31	28
Average	23.49	23.40	24.29
SD	0.44	0.33	0.78
Median	23.45	23.38	24.20
Range	22.25–24.22	22.75–24.00	22.83–26.25
Stnd Skewness	0.31	0.25	1.08
Stnd kurtosis	−0.28	−0.25	0.44
SEM	0.29	0.12	0.30
95% CI	23.20–23.78	23.28–23.52	23.98–24.59

**Table 3:** Descriptive statistics of corneal radius curvature according to refractive status.

Statistics	Refractive status		
	Emmetropes	Hyperopes	Myopes
Count	11	31	28
Average	7.95	7.94	7.69
SD	0.11	0.11	0.16
Range	7.76–8.08	7.67–8.08	7.42–8.04
Stnd skewness	−0.99	−1.89	0.61
Stnd kurtosis	−0.74	−0.71	−1.01
SEM	0.07	0.04	0.06
95% CI	7.88–8.02	7.90–7.98	7.63–7.75

**Table 4:** Descriptive statistics of AL/CRC ratio according to refractive status.

Statistics	Refractive status		
	Emmetropes	Hyperopes	Myopes
Count	11	31	28
Average	2.96	2.95	3.16
SD	0.07	0.07	0.12
Range	2.83–3.04	2.86–3.11	2.94–3.35
Stnd Skewness	−0.75	−2.1	0.11
Stnd kurtosis	−0.80	0.32	−1.2
95% CI	2.91–3.00	2.92–2.97	3.11–3.21
